# Supplementary material for: The RAM signaling pathway links morphology, thermotolerance, and CO2 tolerance in the global fungal pathogen Cryptococcus neoformans
Source: eLife. 2022 Nov 23;11:e82563. doi: 10.7554/eLife.82563 (PMC9708076; doi:10.7554/eLife.82563)
Supplement: Supplementary file 1. [file elife-82563-supp1.docx]

**Supplementary File 1. Hits from forward genetic screening**

| **Gene ID** | **FungiDB Description** | **SGD Name** |
| --- | --- | --- |
| **CNAG_00097** | **myo-inositol transporter, putative** | **ITR1** |
| **CNAG_00293** | **Ras-like protein** | **RSR1** |
| **CNAG_00328** | **DNA excision repair protein ERCC-5** | **RAD2** |
| **CNAG_00333** | **hypothetical protein** | **N/A** |
| **CNAG_00405** | **ste/ste20/ysk protein kinase** | **N/A** |
| **CNAG_00414** | **Maintenance of killer protein 32, putative** | **MAK32** |
| **CNAG_00443** | **hypothetical protein** | **YCR023C** |
| **CNAG_00444** | **hypothetical protein** | **N/A** |
| **CNAG_00458** | **hypothetical protein** | **N/A** |
| **CNAG_00503** | **hypothetical protein** | **N/A** |
| **CNAG_00563** | **hypothetical protein** | **N/A** |
| **CNAG_00577** | **hypothetical protein** | **N/A** |
| **CNAG_00699** | **transmembrane receptor** | **N/A** |
| **CNAG_00833** | **hypothetical protein** | **N/A** |
| **CNAG_00888** | **calcineurin subunit B** | **CNB1** |
| **CNAG_00968** | **hypothetical protein, variant** | **N/A** |
| **CNAG_00991** | **flap endonuclease 1** | **RAD27** |
| **CNAG_01016** | **vacuolar membrane protein** | **MTC5** |
| **CNAG_01038** | **hypothetical protein** | **N/A** |
| **CNAG_01150** | **omega-6 fatty acid desaturase (delta-12 desaturase)** | **N/A** |
| **CNAG_01213** | **hypothetical protein** | **MDM20** |
| **CNAG_01255** | **hypothetical protein** | **N/A** |
| **CNAG_01415** | **cytoplasmic protein** | **YOR296W** |
| **CNAG_01507** | **protein CGI121** | **CGI121** |
| **CNAG_01536** | **myosin heavy chain** | **MYO1** |
| **CNAG_01575** | **ATP-binding cassette transporter** | **YCF1** |
| **CNAG_01653** | **cytokine inducing-glycoprotein** | **N/A** |
| **CNAG_01845** | **AGC/PKC protein kinase** | **PKC1** |
| **CNAG_01864** | **hypothetical protein** | **N/A** |
| **CNAG_01875** | **wd-repeat protein** | **RAV1** |
| **CNAG_01918** | **cytoskeletal regulatory protein binding protein** | **BUD6** |
| **CNAG_01936** | **Sugar transporter** | **N/A** |
| **CNAG_02232** | **RNA polymerase II-associated factor 1** | **PAF1** |
| **CNAG_02332** | **hypothetical protein** | **N/A** |
| **CNAG_02359** | **small subunit ribosomal protein S25e** | **RPS25A** |
| **CNAG_02434** | **Copper transport protein ATX1** | **ATX1** |
| **CNAG_02532** | **D-amino-acid oxidase** | **N/A** |
| **CNAG_02586** | **Sugar transporter** | **N/A** |
| **CNAG_02730** | **sorting nexin-41** | **ATG20** |
| **CNAG_03050** | **hypothetical protein** | **N/A** |
| **CNAG_03155** | **ENTH domain-containing protein** | **YAP1801** |
| **CNAG_03159** | **cytoplasmic protein** | **YAR1** |
| **CNAG_03227** | **hypothetical protein** | **N/A** |
| **CNAG_03301** | **mitochondrial inner membrane translocase subunit TIM13** | **TIM13** |
| **CNAG_03322** | **UDP-glucuronate decarboxylase** | **N/A** |
| **CNAG_03355** | **Two-component-like sensor kinase** | **N/A** |
| **CNAG_03567** | **RAM signaling network protein kinase, putative** | **CBK1** |
| **CNAG_03622** | **cell polarity protein mor2** | **TAO3** |
| **CNAG_03634** | **DNA-directed RNA polymerase I subunit RPA49** | **RPA49** |
| **CNAG_03741** | **hypothetical protein** | **N/A** |
| **CNAG_03745** | **hypothetical protein** | **DAL81** |
| **CNAG_03824** | **solute carrier family 25** | **PIC2** |
| **CNAG_03918** | **ram signaling network protein** | **N/A** |
| **CNAG_03963** | **tyrosine phosphatase** | **OCA1** |
| **CNAG_04048** | **53 kda brg1-associated factor b** | **ARP4** |
| **CNAG_04159** | **ariadne-1** | **HEL1** |
| **CNAG_04243** | **cell division control protein 24** | **CDC24** |
| **CNAG_04351** | **methylmalonate-semialdehyde dehydrogenase (acylating)** | **N/A** |
| **CNAG_04382** | **hypothetical protein** | **N/A** |
| **CNAG_04642** | **tetraspanin Tsp2** | **N/A** |
| **CNAG_04655** | **rab family protein** | **YPT52** |
| **CNAG_04693** | **target of rapamycin complex 2 subunit** | **AVO1** |
| **CNAG_04796** | **calcineurin a catalytic subunit** | **CNA1** |
| **CNAG_04853** | **derlin-2/3** | **N/A** |
| **CNAG_04951** | **3-deoxy-7-phosphoheptulonate synthase** | **ARO4** |
| **CNAG_04992** | **hypothetical protein** | **N/A** |
| **CNAG_05021** | **hypothetical protein, variant** | **REE1** |
| **CNAG_05095** | **pod-specific dehydrogenase SAC25** | **ENV9** |
| **CNAG_05114** | **peroxisomal copper amine oxidase** | **N/A** |
| **CNAG_05159** | **hypothetical protein** | **N/A** |
| **CNAG_05299** | **oxidoreductase** | **N/A** |
| **CNAG_05309** | **hypothetical protein** | **N/A** |
| **CNAG_05451** | **hypothetical protein** | **N/A** |
| **CNAG_05604** | **hypothetical protein** | **N/A** |
| **CNAG_05678** | **membrane protein** | **ADY2** |
| **CNAG_05789** | **hypothetical protein** | **ACL4** |
| **CNAG_05794** | **CBK1 kinase activator protein MOB2** | **MOB2** |
| **CNAG_05882** | **class E vacuolar protein-sorting machinery protein HSE1** | **HSE1** |
| **CNAG_05992** | **hypothetical protein** | **N/A** |
| **CNAG_05998** | **rho family protein** | **N/A** |
| **CNAG_06003** | **hypothetical protein** | **N/A** |
| **CNAG_06218** | **Amidase** | **AMD2** |
| **CNAG_06224** | **nuclear movement protein nudC** | **N/A** |
| **CNAG_06373** | **mitotic spindle assembly checkpoint protein MAD2B** | **REV7** |
| **CNAG_06376** | **vacuolar membrane protein** | **PEP5** |
| **CNAG_06529** | **hypothetical protein** | **N/A** |
| **CNAG_06583** | **hypothetical protein** | **N/A** |
| **CNAG_06589** | **endoribonuclease L-PSP** | **N/A** |
| **CNAG_06664** | **sorting nexin Mvp1** | **MVP1** |
| **CNAG_06716** | **hypothetical protein** | **MUD1** |
| **CNAG_06728** | **kinesin** | **N/A** |
| **CNAG_06796** | **serine/arginine repetitive matrix protein 1** | **N/A** |
| **CNAG_07358** | **hypothetical protein** | **N/A** |
| **CNAG_07438** | **3-keto sterol reductase** | **N/A** |
| **CNAG_07448** | **urea transporter, putative** | **DUR3** |
| **CNAG_07862** | **fumarate reductase** | **FRD1** |
